# Supplementary material for: Are carbon emissions trading and green financial instruments synergistic? -Comprehensive quantitative research based on content analysis
Source: PLoS One. 2024 Mar 7;19(3):e0298601. doi: 10.1371/journal.pone.0298601 (PMC10919601; doi:10.1371/journal.pone.0298601)
Supplement: S1 Data — (ZIP) [file pone.0298601.s001.zip › Data/地级市Z_F工作报告.zip!/║╙─╧/╨┼╤⌠╩╨/└┤╫╘╣1⁄2╓┌║┼í╛┬φ┐╦╩2╛▌═°í┐_2012.docx]

各位代表：

　　现在，我代表市人民政府向大会作工作报告，请予审议，并请各位政协委员和其他列席的同志提出意见。

　　一、过去五年的工作回顾

　　市三届人大一次会议以来的五年，是非常不平凡的五年。市政府在省委、省政府和市委的正确领导下，紧紧围绕“加快三大跨越，建设魅力信阳”的奋斗目标，坚持“四个重在”实践要领，积极用领导方式转变加快发展方式转变，抢抓机遇，应对挑战，团结奋进，真抓实干，战胜了特大洪灾、特大冰雪灾害和严重旱灾，克服了国际金融危机的不利影响，全面完成了三届人大一次会议和“十一五”规划确定的目标任务，经济社会发展呈现出奋进的趋势、跨越的态势、崛起的气势。

　　过去的五年，是我市经济发展总体持续，综合实力跨越提升的五年。我们坚持发展第一要务，突出以产业集聚区发展、项目建设和招商引资三项重点工作带全局，以城乡建设扩内需，以改革创新破难题，国民经济持续保持快速健康发展的良好势头。生产总值保持两位数增长，总量由588.4亿元增加到1276.8亿元，人均突破3000美元，地方公共财政预算收入达到44.3亿元，全部工业增加值达到445.9亿元，社会消费品零售总额实现512亿元，金融机构存款余额达到1266.7亿元，均较2006年翻一番；全市出口总值1.8亿美元，规模以上工业企业实现主营业务收入1285.6亿元、利润67.5亿元，均较2006年翻两番。

　　去年是市三届政府的收官之年，我们较好地完成了全年目标任务，实现了“十二五”良好开局。全市生产总值增长11.1%；地方公共财政预算收入增长30%，增速居全省第3位；规模以上工业增加值增长21.8%，居全省第6位；社会消费品零售总额增长18.2%，居全省第7位；进出口总值增长85.5%，居全省第2位；“双十”工程顺利实施、圆满完成。

　　过去的五年，是我市发展方式加快转变，产业集聚成效显著的五年。我们坚持把产业集聚和转型升级作为加快转变发展方式的重要抓手，扎实推进“一个载体、三个体系”建设，经济发展的协调性、可持续性不断增强。加快产业集聚区建设。突出“三规合一”、“四集一转”、产城融合，集中要素资源，加大扶持力度，产业集聚区基础设施更加完善，主导产业更加明确，综合带动作用更加凸显。规划建设了15个省定产业集聚区，累计完成投资突破1000亿元，入驻规模以上工业企业303家，占全市规模以上工业企业的31%；中心城区以高端制造、新型建材、绿色食品为主导的产业集群初具规模，电子信息产业加快集聚，信阳电子产业园正在崛起，国际家居产业小镇落户信阳；河南省鸡公山文化旅游综合开发试验区、新县省级文化改革发展试验区建设取得显著成效。推动产业结构优化升级。实施了投资千万元以上重大工业转型升级项目437个，已有252个建成投产；规模以上工业增加值占全部工业的比重达到78.5%，提高23.2个百分点，工业化率提高到34.9%；高技术产业规模五年翻两番，年均增长34.8%，高于全市规模以上工业12个百分点。生态经济、劳务经济和商贸物流、金融保险、信息服务业等有了新的发展，二、三产业比重达到74.2%，节能降耗目标圆满完成。全面实施质量立市战略，累计荣获国家级名牌产品1个、中国驰名商标6个、省长质量奖1个、市长质量奖6个，信阳毛尖上升为国家标准。大力推进自主创新体系建设，实施各类科技项目520多项，“豫南稻区籼改粳技术研究与产业化”项目列为省级重大科技专项。

　　过去的五年，是我市改革试验放大提升，“三农”工作稳步推进的五年。我们坚持把“三农”工作摆在重中之重位置，深入推进农村综合改革试验，组建了全省首家农村商业银行、农民专业合作总社和土地信用合作社，“六个放大提升”取得积极进展，“华英农业”富民计划实施富有成效，农村发展活力明显增强。信阳农村改革发展综合试验区写入《国务院关于支持河南省加快建设中原经济区的指导意见》，被中央农村工作领导小组办公室列为农村改革试验联系点。粮食产量连续8年创历史新高，2011年达到115.9亿斤，分别占全国、全省的1/100和1/10强，是全国18个粮食产量超百亿斤的省辖市之一。茶产业迅猛发展，研发生产了信阳红茶，茶园面积扩大到180万亩，浉河茶叶产业集群被省委、省政府确定为全省重点支持的三大农业产业化集群之一。现代农业加快发展，华英农业成功上市，市级以上农业产业化重点龙头企业达到266家，农民专业合作社总量居全省第一。

　　过去的五年，是我市城乡面貌焕然一新，信阳魅力日益彰显的五年。我们坚持推进中心城市、小城市、小城镇和新型农村社区协调发展，编制完成信阳新区建设总体方案，大力实施城乡建设三年大提升行动计划。全市城镇化率达到36.3%，五年提高7个百分点。城市框架不断扩大，中心城区建成区面积由40多平方公里增加到近80平方公里，县城和小城镇建成区面积由295平方公里增加到430平方公里。中心城区建设不断加快，旧城改造有序推进，浉河治理、内河治理、雨污分流、高铁站片区建设等一批重点工程相继实施，百花园区域公益设施建成开放，浉河新八景全面落成，城市形象大大提升。城镇道路、供排水、供电、环保等基础设施不断完善，城镇载体功能日益增强。环境保护和林业生态建设成效显著，“六城联创”成果丰硕，成功创建国家生态示范市、中国优秀旅游城市、国家园林城市、省级文明城市，实现全国“双拥”模范城四连冠，相继获得中国最具投资潜力中小城市、中国创业之城、中国最具绿色竞争力城市、中国最具幸福感城市等多项殊荣，连续三年蝉联中国十佳宜居城市，大大提升了我市的知名度、美誉度和生态软实力。

　　过去的五年，是我市基础设施逐步完善，支撑能力显著增强的五年。我们坚持打基础、积后劲、谋长远、惠民生，全面实施“五网一系统”基础支撑体系建设，全社会固定资产投资五年累计完成4107亿元，年均增长28.1%。一批交通、能源、水利等重大基础设施建成使用，有力支撑了全市经济社会的快速发展。沪陕高速、大广高速相继开通，石武高铁信阳段即将通车，淮息、淮固高速等重点项目快速推进，淮河淮滨至三河尖航运正式通航，信阳明港机场军民合用项目批复立项。全市基本实现县县通高速公路，全部实现村村通水泥（油）路，公路总里程达到2.4万公里，位居全省第二位，成为全国重要的交通枢纽。华豫2×66万千瓦超超临界机组建成投产，全市发电装机容量突破200万千瓦，电网规模、电力输送能力实现翻番。出山店水库前期工作取得重大突破，五大灌区技改和94座水库除险加固工程按计划全部完成。五年完成水利投资39.4亿元，新增有效灌溉面积110万亩，解决农村饮水安全128.2万人。

　　过去的五年，是我市体制机制不断创新，开放格局基本形成的五年。我们坚持以改革创新为动力，以开放带动促发展，不断破解发展难题、赢得发展机遇、拓宽发展空间。深入推进重点领域改革。国有企业改革不断深化，市、县政府机构改革全面完成，事业单位人事工资制度改革全面展开，集体林权制度改革、农村公益事业建设“一事一议”财政奖补试点扎实推进，融资担保体系不断完善，医药卫生体制改革、文化体制改革、供销体制改革、公安警务机制改革有序进行。大力开展招商引资。持续推进“四一”招商行动，积极承接产业转移，狠抓合同履约率、资金到位率和项目落地率，在能源、通信、资源开发、文化旅游、高新技术等重点领域签订了一批战略合作协议，促成了一批重大合作项目，招商引资工作取得历史性突破。五年实际利用外资6.9亿美元，实际利用省外资金300亿元。全面加强区域交流与合作。五年利用外资扩展到17个国家和地区，与63个国家和地区建立了经贸关系，36家国内外500强企业在信阳投资落户，累计外派劳务人员3万多人次。大别山区域合作进一步深化，初步实现优势互补、融合发展。信阳海关获批筹建，“大通关”机制进一步完善。

　　过去的五年，是我市社会事业全面进步，人民得到实惠最多的五年。我们始终坚持改善民生、维护民利，累计投入民生工程525亿元。城镇居民人均可支配收入和农民人均纯收入分别达到15271元和6153元，较2006年接近翻一番。落实国家强农惠农政策，财政惠农补贴达到63.5亿元。积极开发就业岗位，城镇新增就业46.6万人，帮助困难人员就业5.7万人。支持回乡创业，共吸引3万多名成功人士返乡创办各类实体2.8万个，投资总额33亿元，带动就业10.6万人。大力发展社会事业。高中教育质量保持全省领先地位，职教攻坚取得新的进展，信阳农专、信阳职业技术学院、信阳涉外劳务技术学院新校区一期工程和羊山中学建成使用。举办了一系列有影响力的全国性体育赛事，覆盖城乡的公共文化体育设施不断完善，人民群众的精神文化生活更加丰富。基层医疗卫生服务体系基本形成，一批县级医院、乡镇卫生院、社区卫生服务中心投入使用，在全省率先实现所有行政村建有标准化卫生室。统筹人口与计划生育工作，人口自然增长率控制在6‰以内。不断强化社会保障。新农保、新农合实现全覆盖，社会养老保险、城乡低保覆盖面分别扩大3.7倍、12倍，农村五保供养实现应保尽保，最低工资标准和退休人员基本养老金有了较大幅度提高。加快推进保障性住房建设，新建公共租赁住房、廉租住房、经济适用住房31269套。深入推进扶贫开发，又有46.7万人实现脱贫。大力实施“菜篮子”工程，保障了市场物价基本稳定。加强食品药品安全管理，食品药品安全保障水平继续提高。

　　过去的五年，是我市政府建设不断加强，发展环境更加优化的五年。我们坚持“三具两基一抓手”，深入推进“两转两提”，积极开展“创先争优”活动，严格落实党风廉政建设责任制，加强惩治和预防腐败体系建设，政府自身建设迈出新的步伐。自觉接受人大及其常委会的法律监督、工作监督和政协民主监督，认真办理人大代表建议和政协提案。深化行政审批制度改革，政府公共服务集成管理水平全面提升，行政许可事项减少到162项，为全省最少的市之一。坚持依法行政，强化行政监察和审计监督，政务公开深入推进，行政复议、行政执法监督工作继续加强，“五五”普法和依法治理任务圆满完成。积极创新社会管理，严格公众参与、专家论证、风险评估、合法性审查和集体讨论等民主决策程序，大力推行信访工作“四家工作法”，社会治安综合防控体系日益完善，矛盾纠纷调处机制逐步健全，安全生产监管不断加强，社会管理科学化水平不断提升，社会大局和谐稳定。严格执行“五禁止”、“十不准”规定，干部作风建设进一步加强，行政效能进一步提高，发展环境进一步优化。

　　加强精神文明建设，公民道德水平和城乡文明程度明显提升。积极开展“双拥”共建活动，军政军民团结进一步加强，民兵预备役建设取得新进展。驻信解放军和武警、消防部队在支持地方建设、维护社会稳定等方面作出了重要贡献，工会、共青团、妇联和科协、文联、社科联、工商联等人民团体在经济社会发展中发挥了不可替代的作用，民族、宗教、外事、侨务、对台工作全面加强，统计、物价、工商、税务、邮政、无线电管理、机关事务、市场发展、物流管理、地质、气象、地震、人防、档案、史志、残联等工作取得新的成绩。

　　各位代表！

　　经过全市上下的共同努力，经过五年的改革发展，信阳形成了好的定位、好的思路、好的制度、好的氛围，在勇当中原经济区前锋中具有了更加坚实的基础、形成了更加有利的条件、迎来了更加难得的机遇、积蓄了更加强劲的动力。五年取得的辉煌业绩，是全面贯彻落实党的路线方针政策的结果，是省委、省政府和市委正确领导的结果，是市人大、市政协监督支持的结果，是全市人民团结奋斗的结果。在此，我代表市人民政府，向全市广大干部群众、驻信部队、武警官兵、公安干警，向所有关心、支持信阳建设的各界人士表示崇高敬意和衷心感谢！

　　各位代表！

　　五年的探索实践，我们积累了很多宝贵经验：一是必须注重持续求进。坚持“四个重在”实践要领，牢牢把握中原经济区建设的核心任务，持续探索一条不以牺牲农业和粮食、生态和环境为代价，以新型城镇化为引领的“三化”协调科学发展路子，遵循规律，科学运作，乘势而上，带动全局工作上台阶，始终保持跨越发展好的趋势、好的态势和好的气势。二是必须注重统筹协调。统筹改革发展稳定，统筹经济社会发展、城乡发展、区域发展、人与自然和谐发展，统筹各项事业，统筹协调各方力量，形成推动经济社会可持续发展的良性机制和强大合力。三是必须注重务实作为。坚持说了就办，定了就干，求实求效，善做善成，以务实发展树立务实形象，成就了信阳今天蓬勃发展的局面，让信阳更富活力、更具魅力。四是必须注重利民惠民。坚持把执政为民作为政府工作的出发点和落脚点，全面落实惠民政策，不断加大民生投入，大力实施利民工程，办成了一批具有深远意义的大事，办好了一批深受群众欢迎的实事，办妥了一批社会关注的难事，赢得了人民群众的信任和支持。

　　各位代表！

　　在肯定成绩的同时，我们也清醒地看到，我市经济社会发展仍然面临不少矛盾和问题：经济增长的质量效益仍不理想，持续增长的难度逐步加大；三次产业、三大需求之间结构还不协调，城乡、县区之间发展还不平衡，结构性矛盾仍很突出；城镇化、工业化水平不高，仍然是制约发展的短板，跨越发展的基础还不牢固；财政保障能力与经济社会发展之间的矛盾还比较突出，改善民生的任务依然艰巨；一些部门的职能转变还不到位，政府自身建设有待加强。对此，我们一定高度重视，认真加以解决。

　　二、今后五年的目标任务

　　今后五年，是我市圆满完成“十二五”规划、全面提升魅力信阳建设水平的重要时期，也是加快新型“三化”协调发展、全面建设小康社会的关键时期。随着建设中原经济区战略的全面实施，信阳在中原经济区中的战略定位更加清晰，在全国的综合交通和现代物流枢纽地位更加巩固，在深化改革、扩大开放、承接转移中的前沿优势更加凸显。面对新形势、新要求、新使命，我们必须抢抓新机遇，发挥新优势，谋划新蓝图，开创新局面。

　　按照市四次党代会提出的指导思想，今后五年政府工作的总体要求是：深入贯彻落实科学发展观，坚持“四个重在”实践要领，以科学发展为主题，以加快转变经济发展方式为主线，以建设中原经济区区域增长极、战略支撑点和改革试验区为总体战略，以富民强市为目标，持续求进、务实发展、积极作为，加快新型“三化”协调发展，勇当中原经济区前锋，全面提升魅力信阳建设水平。

　　今后五年的战略任务是：全面建设“一市一区两枢纽一基地”，即把信阳建成鄂豫皖三省交界区域性中心城市、国家级农村改革发展综合试验区、全国重要的综合交通枢纽和现代物流枢纽、中部地区承接东南沿海产业转移的重要基地，努力走出一条“两不三新、三化协调”科学发展路子。

　　今后五年的奋斗目标是：

　　——综合实力快速提升。经济持续快速健康发展，粮食综合生产能力不断增强，二三产业比重进一步提高，人口、资源、环境与经济发展更加协调，经济增长的质量效益显著提升，基本形成现代城镇体系、现代产业体系和自主创新体系。力争到2016年，全市生产总值和公共财政预算收入翻一番。

　　——改革开放全面深化。政府效能全面提升，农村改革试验纵深推进，开放型经济快速发展，非公有制经济发展水平大幅提高。发展环境进一步优化，招商引资的质量和水平进一步提升。进出口总额和实际利用外资年均增长30%以上。

　　——生态环境日趋优美。“六城联创”成果得到全面巩固和提升，宜居宜业的特色更加鲜明。水土资源得到有效保护和利用，空气质量好中有升，环境综合整治成效显著，生态经济、循环经济快速发展。林业生态市建设扎实推进，森林覆盖率进一步提高，资源节约、环境友好的生产生活方式加速形成，绿色竞争力持续领先。万元生产总值能耗降低18%。

　　——民生民计持续改善。各项社会事业快速发展，公民思想道德和科学文化素质不断提高。基本公共服务均等化程度进一步提升，广覆盖、保基本、多层次、可持续的社会保障体系基本建立，社会就业更加充分，低收入群体生活质量明显改善，城镇居民人均可支配收入达到2.6万元，农民人均纯收入突破1万元。人民生活更有保障、更有尊严、更加幸福。

　　——社会环境更加和谐。社会建设全面加强，精神文明、政治文明和民主法制建设取得明显成效，社会风气、社会信用和社会秩序进一步优化，社会公平正义充分彰显。社会管理创新能力进一步提高，应急管理机制更加完善，基层社会管理体制逐步健全，社会组织作用充分发挥，人民群众的利益诉求表达渠道更加畅通。公共安全体系更加完备，平安建设扎实推进，群众安全感显著提升，社会大局和谐稳定。

　　实现上述目标和任务，我们必须按照市四次党代会提出的建设“五地”的总体要求，主动融入中原经济区建设，加快推进新型“三化”协调发展。

　　（一）突出新型城镇化引领作用，打造战略支撑点。立足于建设“生态枢纽城”，放大信阳交通区位和生态优势，发挥中心城市龙头带动作用，扎实推进“五网一系统”基础支撑体系建设，进一步提升“山水信阳、休闲茶都”品牌，彰显信阳宜居宜业宜游和最具幸福感城市特色，构建中心城市、小城市、小城镇、新型农村社区协调发展、互促共进的新型城镇化体系，力争城镇化率达到50%左右，打造沿宁西轴线城镇连绵带，把信阳加快建成鄂豫皖交界区域性中心城市和中原经济区南部重要的战略支撑点。立足于建设全国综合交通和现代物流枢纽，着力构建便捷、通畅、高效、安全的现代综合交通体系，增强服务中原经济区建设的支撑保障能力，努力打造中心城区至各县区“一小时交通圈”、县城至各乡镇“半小时交通圈”；推进现代物流业跨越式发展，加快大型物流企业建设，培育大型物流内陆港，构建现代物流网络。立足于建设中部地区文化旅游中心，以特色文化引领县域经济发展，提升红色文化、根亲文化、茶文化、民俗文化等文化软实力，以文化涵养城市，促进文化大发展大繁荣；发展壮大鸡公山文化旅游产业集聚区，推进文化、旅游融合发展，打造中国国际山地旅游休闲度假目的地；加强与周边省市区域合作，规划建设大别山旅游区。

　　（二）突出新型工业化主导作用，打造区域增长极。立足于建设“特色集聚区”，坚持“三规合一”、“四集一转”，育龙头、建基地、抓配套，不断提升产业集聚区建设水平。立足于建设中部地区承接东南沿海产业转移的重要基地，突出主导产业结构升级，围绕产业链条图谱招商，加大链式承接、组团承接、板块承接和差异化承接，发展壮大高端制造、绿色食品、新型建材、电子信息、商贸物流、现代家居等特色产业集群，努力创建中原经济区新型工业化示范城市，加快把信阳打造成为全省重要的电子信息产品制造基地和中部地区家居研发生产销售中心，形成区域经济增长极。突出产业为基、就业为本、产城融合，支持每个县分别建设一个产业集聚区、一个人口规模达到20万的县城和一个产值超百亿元的主导产业。

　　（三）突出新型农业现代化基础作用，打造改革试验区。立足于建设国家级改革试验先行区，持续深化信阳农村改革发展综合试验区建设，提升改革试验层次，强化“六个放大提升”，着力在农村土地流转、金融创新、专业合作、社会保障、现代农业产业化等方面，破解好“人往哪里去、钱从哪里来、粮食怎么保、民生怎么办”四大难题，为全省乃至全国探索路子、积累经验。立足于建设“绿色农业谷”，打造特色农业示范园区和生产基地，实施粮食高产创建工程和现代农业产业化富民工程，大力发展绿色农业和生态农业。争取水环境质量生态补偿政策，落实淮河源生态补偿机制，推行排污权有偿使用和交易。持续推进林业生态市和淮河源生态功能保护区建设，加强天然林、涵养林、湿地和生物多样性保护，增强信阳可持续发展能力。

　　各位代表！

　　我们坚信，只要全市上下众志成城，朝着既定目标奋勇前进，一个经济更有竞争力、社会更有创新力、文化更有影响力、环境更有吸引力、百姓更有自豪感的崭新信阳，必将以多姿多彩的魅力展现在世人面前、屹立在中原经济区前沿！

　　三、关于2012年工作安排

　　今年是中原经济区建设的关键之年，也是本届政府的开局之年，做好今年工作意义重大。上半年，我们紧紧抓住中原经济区战略全面实施的重大机遇，着力扩需求、创优势、破瓶颈、惠民生，全市经济社会发展持续了总体向好的基本态势。但是，受国内外复杂形势影响，经济运行的下行压力加大，总体形势依然严峻，我们必须深谋远虑抓大事，千方百计解难事，雷厉风行办急事。

　　今年主要预期目标是：地区生产总值增长11%，公共财政预算收入增长12%以上，全社会固定资产投资增长21%，社会消费品零售总额增长16%，外贸出口增长30%，实际利用外商直接投资增长30%，引进省外资金增长30%，城镇居民人均可支配收入实际增长9%左右，农民人均纯收入实际增长9%左右，居民消费价格涨幅控制在4%以内，人口自然增长率控制在6.5‰以内，节能减排完成省下达任务。

　　实现上述目标，重点做好以下工作。

　　（一）着力强化城乡统筹，持续推进新型城镇化进程

　　构建现代城镇体系。坚持以新型城镇化为引领，全面融入中原经济区建设。增强中心城市辐射带动作用，加快城市新区、中心城市组团、老城区改造三大板块建设，统筹规划商务中心区和特色商业区（街），推进浉河、平桥、羊山、工业城、上天梯、南湾六区联动、一体发展，抓好180个重大城市建设项目，年度完成投资130亿元以上。提升县城承载作用，落实省直管县政策，加快潢光一体化进程，推进固始、潢川向区域性中心城市发展。突出产城融合，支持县区重点抓好城市新区开发、老城改造、产业集聚区发展，不断提升县域经济综合实力。认真落实中央编办等六部委《关于开展经济发达镇行政管理体制改革试点工作的通知》精神，将明港提升到小城市层面加快发展。发挥乡镇承接作用，启动新型城镇化引领示范乡建设，实施130个重大项目，总投资50亿元。进一步完善进城务工人员和纳入新型社区人员的就业、安居、子女入学、社会保障、户籍管理等机制，加快农村人口有序转移。积极推进以公共租赁住房建设为主的保障性安居工程建设，促进房地产市场健康发展。

　　提升城镇载体功能。加快明港机场前期工作，谋划推进明港—天目山—出山店水库—南湾湖—鸡公山环城高速项目，开工建设中石化驻马店至信阳成品油管道及下载库工程，加快高铁站片区建设，实施107国道、216省道信阳段等公路升级改造，改扩建三河尖望岗码头，启动建设淮滨“公铁水一体化”项目。统筹全市交通规划，优化中心城区“五纵六横”交通路网格局，坚持公交优先，促进公交与各种交通系统衔接，提升公共交通整体服务水平。加快中心城市“畅通工程”、地下空间开发利用和基础设施配套，继续加强浉河治理，推进内河治理、雨污分流、污水处理、中水回用、垃圾处理等项目建设，加大中心城区市场建设步伐，实施中心城区城中村、集中连片棚户区改造，加快推进新一轮电力城农网改造工程。加强城市数字化、精细化管理，深入推进“六城联创”，完成创建国家卫生城市主要任务。

　　积极推进新型农村社区建设。把建设新型农村社区作为推进“三化”协调发展的战略基点，坚持“分类指导、科学规划、群众自愿、就业为本、量力而行、尽力而为”原则，科学合理确定新型农村社区布局、规模和建设标准，积极推进新型农村社区基础设施和公共服务设施先行先建，引导农户不在没有规划的地方和基础设施不配套的地方建房，严禁沿路乱搭乱建。加快实施30个市级新型农村社区建设试点，整合要素资源，完善基础设施，推进村民自治，强化产业支撑，有效推动土地集约利用、农业规模经营、农民多元就业。

　　（二）着力强化产业集聚，持续提升新型工业化水平

　　打造现代产业体系。重点围绕高端制造、新型建材、绿色食品三大战略支撑产业和电子信息、现代家居两大高成长性产业，培育一批特色产业集聚区和专业园区。高端制造方面，重点抓好航天精工、同合车轮、贝恩银光、万华制造、淮滨造船、舜宇光学等重点项目和专业园区建设；新型建材方面，重点依托上天梯产业集聚区，进一步提升新型节能保温建材产业集聚水平；绿色食品方面，重点发展茶叶加工、畜禽加工、粮油加工三大集群产业，加快实施一批农业产业化集群项目；电子信息方面，强力推进激蓝OLED项目建设，抓好泛蓝科技、天扬光电、东信蓝光、天恒液晶、星天空数码、博仕达通信和台商电子产业园等重点项目，为把信阳工业城建成全省重要的新型工业化示范基地创造条件；家居产业方面，加快实施羊山新区国际家居小镇项目，力争早日进入全省产业集聚区行列。围绕构建中原经济区南部商贸物流和文化旅游“两大中心”，加快潢川开发区物流园区、金牛物流园区、鸡公山文化旅游综合开发试验区发展，推进义乌国际小商品城、国际商贸城、国际汽车城、信阳国际茶城等重大项目建设。发展壮大生物医药、新能源汽车两大战略先导产业，突出抓好新县产业集聚区生物医药产业园、信阳市产业集聚区新能源汽车产业园建设。改造升级钢铁和纺织两大传统优势产业，力争信钢集团主营业务收入突破150亿元，规模以上纺织服装企业达到100家。

　　做强集聚发展载体。继续强化落实验收、督导、观摩机制，重点围绕产业集聚区建设，突出主导产业，延伸、拓展产业链条，推进项目择地落户，打造特色产业集群。力争规模超100亿元的产业集群达到2个，超50亿元的产业集群达到5个。适时推进产业集聚区由建设服务向生产服务转变、项目配套向生活配套转变、发展保障向社会保障转变，力争全年完成基础设施投资150亿元左右，修建道路160公里、供排水管道300公里，建设标准化厂房130万平方米，建成公共租赁住房6000套以上，规划建设一批中介服务机构和商业、文化娱乐、医疗等配套设施。

　　提升集约发展水平。以优势企业为核心，加强产学研合作，组织实施一批重大科技专项，提升企业自主创新水平。实行强度和总量双控制，加强产业准入管理，大力淘汰落后产能，认真开展企业节能专项行动计划，促进环境容量高效利用和环境资源优化配置。

　　（三）着力强化改革试验，持续加快新型农业现代化步伐

　　推进高标准粮田“百千万”工程。落实国家千亿斤粮食战略工程和省粮食生产核心区规划，启动建设一批百亩方、千亩方和万亩方高标准永久性粮田，实施水稻百亩争创工程和省重大科技专项豫南水稻籼改粳工程。在粮食主产区建设高产稳产粮田22万亩以上，改造中低产田8万亩以上，建设高标准粮田7万亩左右，实施土地整理和复垦开发5万亩以上。积极发展生态农业，完成农田防护林体系改扩建工程1.3万亩。加强水利基础设施建设，继续做好大中型灌区续建配套和节水改造，实施中小河流治理和淮河流域重点平原洼地治理。加快中小型病险水库除险加固，加大塘堰清淤力度，推进小农水重点县建设，抓好防汛抗旱工作，提高农业抗灾减灾能力，确保粮食总产稳定在110亿斤以上。

　　推进农业产业化集群培育工程。加大政策和资金支持力度，培育壮大农业产业化龙头企业，重点建设浉河茶叶、平桥现代牧业、固始三高农牧、潢川花木、光山羽绒、华英畜禽、黄国粮业、万富油脂、豫申粮油、商城山信、息淮弱筋小麦、信阳油茶和中药材等农业产业化基地，着力打造“全链接、全循环、高质量、高效益”现代农业产业集群。统筹推进田间生产设施配套建设，大力发展特色农业和观光农业，建立健全现代农业服务体系，支撑和保障农业产业化发展。

　　推进主食产业化工程。充分发挥我市粮油资源优势，做大做强主食加工龙头企业，大力发展以主食为重点的粮油深加工，积极推进主食产业集群和粮油加工园区建设，建成一批标准化主食专用水稻生产基地和专用小麦生产基地，构建从田间到餐桌的粮食产业全产业链发展模式，培育具有信阳特色的主食优势产品，推动信阳特色农副产品组团形成整体优势。

　　推进农村综合试验区建设。围绕“六个放大提升”，持续在完善土地承包经营权、创新现代农业经营机制、提高农村金融服务水平、健全农村社会保障制度等方面积极探索，大力推动农民、农地、农机、农艺“四农联动”，进一步完善以农村“五权”确权为主的农村资产、用益物权抵押担保机制，促进农民增收、农业增效、农村发展，巩固和发展“三农”工作好形势。

　　推进强农惠农政策落实。坚持财政支出、固定资产投资、土地出让收益重点向农业农村倾斜，确保用于农业农村的总量、增量、增幅、比重均有提高。积极引导金融机构加大涉农信贷投放，确保涉农信贷增量占比不低于上年。及时兑现农民种粮各项补贴，加大对农业政策性保险支持力度，调动农民发展农业生产积极性。继续实施村级公益事业建设“一事一议”财政奖补政策。积极整合涉农资金，大力实施大别山连片扶贫开发和整村推进项目。做好淮干滩区居民迁建和安置区配套建设。

　　（四）着力强化招商引资，持续扩大对外开放成果

　　提升开放水平。科学运用“五个三”招商方法，深化“四一”招商行动，突出抓好产业集聚区招商、与央企合作和承接产业转移，跟踪落实第二十届国际茶文化节等重大招商活动签约项目，不断扩大开放招商成果。突出招大引强，发挥招商“四库”作用，紧盯国内外500强和行业知名企业，推进落实与中石油、中石化、国电集团、江苏雨润等央企的签约项目，加强与港中旅、上文广、中粮、百威英博等集团的战略合作，再引进和实施一批重大合作项目。扎实推进信阳茶叶、宏润冷冻、固始柳编、樱桃谷鸭、豫南黑猪、中川水产、甾体生物、光山羽绒、瑞孚实业等出口产业基地建设，积极申报综合保税区、出口加工区和保税仓库，加快信阳海关建设。同时，大力引进劳动密集型产业，为扩大就业和农民向城镇转移创造条件。

　　拓宽开放领域。加快城乡建设、农业水利、科教文卫、服务业等领域开放步伐，吸引更多境内外资金进入高技术产业、现代服务业和现代农业，参与城镇化和农村基础设施建设。积极开展金融招商，吸引境内外各类金融机构和各种基金、金融中介组织来我市设立分支机构，鼓励市外、境外资金参股本地法人金融机构。围绕新型“三化”协调发展，科学谋划一批对外招商项目，促进多元化投资、产业化经营。

　　完善开放机制。全面落实对外开放基本省策，形成举市开放长效机制。建立科学评估体系，将招商项目在产业集聚区的落地率、投入产出率、就业和税收贡献率等作为重要评价指标，严格督导考核，确保每个县区年内新上1－3个亿元以上产业项目。继续开展投资环境评价评议活动，严肃查处损害经济发展环境行为，营造亲商、安商、富商的良好氛围，推动对外开放实现更高水平、更大规模、更好效果。

　　（五）着力强化项目带动，持续夯实跨越发展基础

　　深入开展“招商引资项目落地年”活动，持续提高工业项目、产业集聚区项目和大项目占重点项目投资的比重，带动全社会固定资产投资突破1200亿元。提升项目谋划水平。抢抓中原经济区建设、产业转移及国家扩大内需政策等重大机遇，认真谋划、储备一批支撑、辐射、带动作用强的基础设施和主导产业项目，加强科学运作，力争更多项目进入国家、省项目笼子。加快项目建设进度。坚持“有限资源保重点、集中力量办大事”，加强要素保障，确保重点项目建设有效推进。大力实施淮息淮固高速公路、西气东输二线、弘昌燃气发电等106个省市重点项目，加快推进信阳电子产业园、国际家居产业小镇、茶产业园等十大项目和149个重点工业项目，积极实施一批城乡建设、基础设施和社会事业项目，促进全社会固定资产投资增长。强化项目推进机制。坚持领导联系重点项目、会审会签、联审联批制度，继续强化产业集聚区“四集中”和“零阻碍”服务，完善项目谋划、招商、落地和服务四项工作机制，加大跟踪协调服务力度，确保项目早开工、早投产、早见效。

　　（六）着力强化机制创新，持续增强经济发展活力

　　深入推进国有企业改革重组，完善企业法人治理结构，基本完成市属企业脱钩改制工作。加快非公有制经济发展，落实支持民营经济和中小型、微型企业发展的各项政策措施，鼓励和引导民间资本通过多种方式进入基础设施、公用事业、保障性住房和社会事业等领域，参与国有企业重组。深化财税金融体制机制改革创新，争创信阳农村金融改革试点市，积极支持信阳银行做强做大，加快发展村镇银行、小额贷款公司等新型金融机构，大力支持企业上市融资，鼓励保险资金参与基础设施建设，着力拓宽区域融资渠道，完善金融服务体系，优化金融生态环境。创新土地管理机制，实施差别化土地政策，切实保障农民合法土地权益，实现土地高效利用。深入推进事业单位分类改革、集体林权制度改革、农村信用社改革、农电体制改革，加快县级供销社体制改革，继续深化文化体制、医药卫生体制、警务机制改革创新。积极争取上级出台支持大别山地区经济社会发展优惠政策。

　　（七）着力强化生态建设，持续积聚区域竞争优势

　　强力推进节能减排，严禁引进“两高一低”项目，确保实现节能减排目标。全面推广清洁生产，着力创建循环经济示范县（区）、示范产业集聚区，培育一批零排放示范企业。切实加强环境保护，继续推进重点流域、重点区域、重点行业污染综合整治，加强淮河源国家重点生态功能区保护，逐步探索建立淮河源生态补偿机制和水环境质量生态补偿机制。大力实施天然林保护和林业生态建设工程，启动“全国绿化模范城市”创建工作。加快“南湾水库国家湖泊生态环境保护试点”项目建设，重点整治南湾湖饮用水源地畜禽养殖和周边乡镇生活污染问题，保护好城市饮用水水源地。加强农村环境综合整治，建设一批环境优美乡镇和生态村，全面提升生态文明水平。

　　（八）着力强化民生建设，持续促进社会和谐稳定

　　加大民生投入，着力提高基本公共服务均等化程度，大力发展各项民生事业。千方百计扩大就业。继续实施积极的就业政策，多渠道开发就业岗位。实施农村劳动力技能培训、全民技能振兴工程，实施公益性岗位安置计划，持续壮大劳务经济，大力发展“农家乐”旅游，确保每个零就业家庭至少有一人就业，稳定提高低收入群体生活水平。积极开展劳动监察执法活动，维护劳动者合法权益。完善社会保障体系。积极推进城镇职工医保、城镇居民医保、新农合、城乡医疗救助四项制度有序衔接，规范完善失业、医疗、工伤、生育保险市级统筹，加强社会救助体系建设，继续增加企业退休人员基本养老金，提高城乡低保补助水平、农村五保供养水平、困难群众大病救助水平和优抚对象保障标准，争取成为全国城乡困难群众大病救助试点。加快推进河南省离退休人员养老基地和工伤预防康复基地前期工作。大力发展社会福利和慈善事业、妇女儿童事业、老龄事业、残疾人事业，启动实施关爱留守儿童和空巢老人工程。加快教育改革发展。加快教育资源优化整合，积极推进学前教育三年行动计划，完善义务教育经费保障机制，优化中小学校布局，改善城区办学条件，推进义务教育均衡发展。加快信阳职教园区和平桥公共实训基地建设，打造一批省级职教园区、示范性职业院校和优质特色职业院校。提高医疗保障水平。加强基层医疗卫生服务体系建设，继续免费提供居民健康教育、妇幼保健、计划免疫等基本公共卫生服务，抓好艾滋病等重大传染病防控与救治工作。加强对蜱虫病的研究和防控治疗，提高防治效果。统筹做好人口和计划生育工作，稳定低生育水平。大力振兴文化体育。扎实推进鸡公山文化旅游综合开发试验区和新县文化改革发展试验区建设，逐步完善县区图书馆、博物馆、文化馆、体育馆和青少年活动中心等文体设施，大力实施乡镇综合文化站、农家书屋、农村电影放映、广播电视村村通等惠民工程，深入开展全民健身活动。推进社会管理创新。健全“党委领导、政府负责、社会协同、公众参与”的社会管理格局，强化基层社会服务管理功能，提升社会管理科学化水平。深入实施“质量立市”战略，严格食品药品安全监管，抓好“菜篮子”工程和食品安全“四大放心工程”。建立重大事项风险评估机制，提高对自然灾害、事故灾难和重大公共安全等事件的应急处置能力。建立覆盖全社会的征信系统，推进社会信用体系建设。大力开展“安全信阳”创建活动，加强重点领域安全监管。深入实施“六五”普法和依法治理规划，加强法制宣传和法律援助，提升信访工作“四家工作法”，积极探索多元化社会矛盾纠纷调处机制，加强社会治安综合治理，依法打击各种违法犯罪活动，维护社会大局和谐稳定。

　　积极支持国防和军队建设。抓好民兵预备役建设和人民防空工作，加强国防教育，增强全民国防意识。认真落实优抚安置政策，巩固和发展军政军民团结。支持工会、共青团、妇联和科协、文联、社科联、工商联等人民团体发挥桥梁纽带作用，全面落实民族、宗教政策，切实做好外事、侨务、对台工作。继续抓好统计、物价、工商、税务、邮政、通信、检验检疫、无线电管理、机关事务、市场发展、物流管理、地质、气象、地震、档案、史志等各项工作。

　　今年，在完成省定“十项重点民生工程”的同时，还将继续抓好我市“双十”工程。2012年“双十”工程安排意见已经印发，请予审议。

　　各位代表！

　　无论是今后五年谋划，还是今年工作安排，都持续已往好的思路和作风，凝聚各方的智慧和共识。我们坚信，只要坚持一张蓝图绘到底，咬定青山不放松，用事业汇聚力量，靠实干谱写华章，就一定能够推进魅力信阳建设再上新台阶！

　　四、全面加强政府自身建设

　　做好今年乃至今后五年的政府工作，必须坚持以领导方式转变加快发展方式转变，认真落实“三具两基一抓手”，深入推进“两转两提”，建设务实政府，树立务实形象，切实做到对人民负责、受人民监督、让人民满意。

　　（一）坚持依法行政。深入贯彻落实《国务院关于加强法治政府建设的意见》，严格依照法定权限和程序行使权力、履行职责，把依法行政贯穿于政府工作全过程。坚持重大事项集体研究、集体决策，广纳民意、广集民智，提高政府决策的科学性、民主性和规范性。严格执行人大及其常委会的决议决定，主动接受其法律监督和工作监督，自觉接受政协民主监督，广泛听取民主党派、工商联、无党派人士和各人民团体的意见建议，认真办理人大代表建议和政协提案。强化政府内部监督，重视舆论和人民群众监督。加强行政复议工作，强化行政执法责任和过错追究，推进严格执法、公正执法、文明执法。不断加大政务公开和政府信息公开力度，畅通政府与公众互动渠道，提高政府工作透明度，切实做到政府职权在阳光下运行、政府工作在监督中推进、政府公信力在民意中提升！

　　（二）强化务实勤政。坚持把务实发展作为一种追求、一种品格、一种责任。不断完善民主决策机制，努力使政府的重大决策、重大部署符合信阳实际、符合群众意愿。坚持“学明白、想明白、说明白、做明白”，责随职走，心随责走，遵循规律，科学运作，创新方法，推动落实。坚决治理庸、懒、散、软等问题，精简会议文件，改进会风文风，减少迎来送往，腾出更多精力用于发展经济、改善民生。健全重大事项、重点工作跟踪落实机制，完善绩效考核指标体系和激励约束评价机制，确保政令畅通、令行禁止。积极开展争做“人民满意的公务员”活动，加强党员干部先进性、纯洁性教育，提素质、强能力、增效率、树形象，努力建设一支政治坚定、业务精湛、作风过硬、人民满意的公务员队伍。

　　（三）持续惠民为政。坚持以人为本，牢记为民宗旨，加快政府职能转变，加强政府公共服务体系建设，切实从群众最关心、最直接、最现实的问题入手，从企业发展最需要、最迫切、最突出的问题入手，真心实意解难题、办实事。逐步提高民生支出比重，使公共财政持续向民生事业、向基层延伸，让人民群众更多地分享发展成果。深入治理征地拆迁、住房保障、食品药品安全、环境保护、安全生产等方面的突出问题，维护人民群众合法权益。

　　（四）严格廉洁从政。认真落实党风廉政建设责任制，扎实推进惩治和预防腐败体系建设，深入开展反腐倡廉宣传教育，积极推进反腐倡廉制度创新，不断拓展从源头上预防腐败工作领域。严格执行“五禁止”、“十不准”和廉洁从政的各项规定，加大纠风和专项治理力度，强化重点领域、关键环节的经济责任审计和财政风险防控，不断加强对权力运行的制约和监督。坚持勤俭节约，制止铺张浪费，严格控制行政经费和一般性支出，努力营造风清气正、廉洁高效的政务环境。严肃查处违纪违法案件，依法严惩腐败分子，切实解决损害群众利益的突出问题，以廉政建设和反腐败斗争实效保障发展、取信于民。

　　各位代表！

　　回首过去，我们负重拼搏、加压奋进；展望未来，我们豪情满怀、稳步跨越！让我们紧密团结在以胡锦涛同志为总书记的党中央周围，认真学习贯彻好即将召开的党的十八大精神，在省委、省政府和中共信阳市委的正确领导下，在全面推进中原经济区建设的大潮中，牢记使命，坚定信心，务实进取，开拓创新，勇当中原经济区前锋，全面提升魅力信阳建设水平，为实现信阳经济社会又好又快发展作出新的更大的贡献！
